# Association between serum uric acid and urinary uric acid excretion indicators and early impaired kidney function in newly diagnosed T2DM patients with normal serum uric acid levels: a retrospective study

**DOI:** 10.7717/peerj.20881

**Published:** 2026-02-27

**Authors:** Xinhang Li, Xiaodan Zhang

**Affiliations:** 1 Department of Endocrinology, The Second Affiliated Hospital, Guangzhou Medical University, Guangzhou, China; 2 Department of Emergency, The Second Affiliated Hospital of Air Force Military Medical University, Xi’an, China

**Keywords:** Type 2 diabetes mellitus, Uric acid, Early impaired kidney function

## Abstract

**Objective:**

To analyze the association between serum uric acid (SUA), urinary uric acid excretion indicators and early impaired kidney function (EIKF) in newly diagnosed type 2 diabetes (T2DM) patients with normal SUA levels, and to analyze their potential value in predicting the risk of EIKF.

**Methods:**

A cross-sectional study was conducted in 628 patients with newly diagnosed T2DM who were admitted to ward of endocrinology department. According to the renal function, patients were divided into non-EIKF group (estimated glomerular filtration rate (eGFR) ≥ 90 ml/min/1.73 m^2^ and urinary albumin-to-creatinine ratio (UACR) < 30 mg/g) and EIKF group (eGFR < 90 ml/min/1.73 m^2^ and (or) UACR ≥ 30 mg/g). Anthropometric, clinical, and biochemical data were collected. Regression analysis was conducted to investigate the associated factors of EIKF. Receiver operating characteristic (ROC) were constructed to assess the potential detective value of the uric acid variables on EIKF risk.

**Results:**

Compared with non-EIKF group, patients in EIKF group were older and had a higher proportion of hypertension. Systolic blood pressure (SBP), HOMA-β, HOMA-IR, SUA, UACR, fractional excretion of uric acid (FEur), and estimated uric acid glomerular filtration (EurGF) levels were higher, while urinary uric acid (Uur), *uric acid clearance* (Cur) and glomerular filtration load of uric acid (FLur) levels were lower in patients with EIKF. Multivariate logistic regression analysis showed that age, SBP and EurGF were associated with the prevalence of EIKF. The ROC curve analyses revealed high areas under the curves of EurGF or the combination of SUA and FEur for detecting EIKF.

**Conclusion:**

For newly diagnosed T2DM patients, even SUA levels are in the normal range, higher SUA, FEur and EurGF are associated with EIKF.

## Introduction

It is widely accepted that moderately increased albuminuria is not a sensitive indicator for the initial clinical manifestation of diabetic nephropathy (Said & Nasr, 2016). Therefore, the recognition of early impaired kidney function (EIKF) is crucial in patients with diabetes. Serum uric acid (SUA) is the end product of purine degradation. Uric acid functions as a powerful antioxidant by effectively neutralizing singlet oxygen, oxygen radicals, and peroxynitrite (ONOO^−^) through its ability to donate electrons, thereby acting as a potent reducing agent. It readily donates hydrogen atoms to free radicals, stabilizing them and preventing further oxidative damage. However, at elevated intracellular concentrations, uric acid can act as a pro-oxidant by activating NADPH oxidase, an enzyme involved in the generation of reactive oxygen species. In addition, uric acid exhibits pro-inflammatory properties, modulates nitric oxide levels, influences immune system interactions, and possesses anti-aging effects (Du et al., 2024). Hyperuricemia is associated with a range of conditions, including hypertension, stroke, obesity, metabolic syndrome, insulin resistance, type 2 diabetes (T2DM), dyslipidemia, and cardiovascular diseases (Copur, Demiray & Kanbay, 2022; So & Thorens, 2010). Previous studies have demonstrated that hyperuricemia predicts the progression of diabetic kidney disease in T2DM patients (Zhu et al., 2023). Nevertheless, lowering SUA levels has not been proven to delay the progression of kidney damage (Badve, 2020; Siu et al., 2006). Interestingly, some studies have shown that both hypouricemia (<120 µmol/L) and hyperuricemia are associated with EIKF in newly diagnosed T2DM patients (Pineda et al., 2019). In a prospective study, Seguro et al. (2015) found that SUA levels exhibit a U-shaped association with kidney function decline. Moreover, prior research indicates that SUA concentrations in the high-normal range are linked to impaired renal function in patients with type 1 diabetes (Ficociello et al., 2010), highlighting the need to examine the relationship between normal uric acid levels and EIKF. However, the risk of EIKF development remains unclear in newly diagnosed T2DM patients with normal SUA levels.

Under physiological conditions, uric acid metabolism occurs primarily in the liver, kidneys, and intestines. Circulating urate levels are tightly regulated by the balance between production and excretion, with the kidneys playing a central role in maintaining this homeostasis (Du et al., 2024). It is recognized that defective renal handling of uric acid accounts for approximately 90% of hyperuricemia and gout cases, due to factors such as reduced glomerular filtration rate (GFR), enhanced tubular reabsorption, or insufficient secretion (Kannangara et al., 2012; Tin et al., 2019). Consequently, alterations in uric acid excretion warrant attention in the context of impaired kidney function. Multiple urinary uric acid excretion indicators exist, including 24-hour urinary uric acid excretion (Uur), fractional excretion of uric acid (FEur), uric acid clearance rate (Cur), glomerular filtration load of uric acid (FLur), excretion of uric acid per volume of glomerular filtration (EurGF), and the urinary uric acid to urinary creatinine ratio (Uur/Ucr). Li et al. (2018) reported that Uur is inversely correlated with albuminuria. Furthermore, Guan et al. (2020) demonstrated that FEur and EurGF increase as renal function declines, while Cur and FLur may serve as more reliable indicators for classifying hyperuricemia in patients with chronic renal failure. However, evidence regarding the characteristics of urinary uric acid excretion parameters in newly diagnosed T2DM patients is limited. In this study, we aimed to evaluate the associations between SUA levels and urinary uric acid excretion indicators with EIKF in newly diagnosed T2DM patients who have normal SUA levels.

## Materials and Methods

### Study population

Data were collected retrospectively from January 2017 to June 2023 at the Second Affiliated Hospital of Guangzhou Medical University. Selection criteria were hospitalized patients with newly diagnosed T2DM. Exclusion criteria were individuals taking any drug that might influence uric acid metabolism, such as diuretics, angiotensin receptor blocker, allopurinol, febuxostat and benzbromarone, or whose clinical data were incomplete. Eventually, a total of 628 patients were included. Patients were admitted due to high glycemic level (HbA1c ≥ 9%) or diabetic complications including acute complications (diabetic ketosis, diabetic ketoacidosis) and chronic complications (diabetic peripheral neuropathy, diabetic foot, *etc.*). This study was approved by the Ethics Committee of the Second Affiliated Hospital of Guangzhou University. The committee waived the need for informed consent due to the retrospective nature of the study with no impact on health outcome.

### Measurement and data collection

Demographic information including history of prescription medications and habits of smoking and drinking were collected through review of medical records. Diabetes was defined according to the guidelines for the prevention and treatment of type 2 Diabetes in China (Chinese Diabetes Society, 2014). Routine biochemical parameters (including fasting plasma glucose (FPG), fasting C-Peptide, glycated hemoglobin (HbA1c), serum high-density lipoprotein cholesterol (HDL-C), low-density lipoprotein cholesterol (LDL-C), total cholesterol (TC), triglyceride (TG), Apolipoprotein A1 (ApoA1), Apolipoprotein B (ApoB), seurm uric acid (SUA), serum creatinine (SCr)) were measured by routine laboratory methods. The estimated glomerular filtration rate (eGFR) was calculated using Modification of Diet in Renal Disease equation: eGFR (ml/min/1.73 m^2^) = 186 × (SCr/88.4)—1.154  × (age) −0.203  × (0.742 if female) (Levey et al., 1999). Homeostatic model assessment of insulin resistance (HOMA-IR) and β cell function (HOMA-β) was calculated using well-established methods: HOMA-IR = 1.5 + fasting blood glucose (mmol/L) × fasting C-peptide (pmol/L)/2800, HOMA-β = 0.27 × fasting C-peptide (pmol/L)/(fasting blood glucose (mmol/L)—3.5) (Li et al., 2004). Hyperuricemia and hypouricemia were defined as SUA levels >420 µmol/L (7.0 mg/dL) and ≤ 120 µmol/L (2 mg/dL), respectively (Li et al., 2004). Serum uric acid levels of the selected study population were defined as in the normal range, (120 µmol/L (2 mg/dL) <SUA ≤ 420 µmol/L (7.0 mg/dL)). Urinary albumin levels were measured from spot urine samples using nephelometry immunoassay and urinary creatinine levels were measured using velocity method. The urinary albumin-to-creatinine ratio (UACR) was calculated. Each participant was instructed to collect the urinary sample correctly. They were provided with a plastic bucket, following settled steps. The collection of 24-hour urine must be done by discarding the first morning void and collecting all urine output for the next 24 h, including the first morning void the next day. There were no dietary restrictions. Well-trained nurses were responsible for recording the start and end of each specimen collection. Uur (µmol/24h) were obtained on the same day as the 24-hour urine sample collection. Creatinine clearance (Ccr, ml/min) was calculated as urine creatinine concentration ×24-hour urine volume (UV)/SCr. FEur (%) was calculated as (Uur × SCr)/(SUA × Ucr) ×100, expressed as percentage. Cur (ml/min) was calculated as Uur × UV/SUA. FLur (µmol/min) was calculated as Ccr × SUA. EurGF (µmol/L) was calculated as (Uur × SCr)/Ucr. EIKF was defined as eGFR <90 ml/min/1.73 m^2^ and/or UACR ≥ 30 mg/g. Non-EIKF was defined as eGFR ≥ 90 ml/min/1.73 m^2^ and UACR <30 mg/g (Joo et al., 2020).

### Statistical analysis

Normally distributed continuous values were presented as mean ± standard deviation (SD). Non-normally distributed continuous data were presented as median (interquartile range). Categorical variables were presented as number (%). Comparisons for continuous variables were performed using *t*-test or Mann–Whitney U test. One-way analysis of variance (ANOVA) was used to compare multiple continuous variables. Categorical variables were compared using Chi-square test. The associations between variables were evaluated by Spearman correlation coefficient test. Correlations between SUA, urinary uric acid excretion indicators and EIKF were analyzed with by multivariate logistic regression. Receiver operating characteristic (ROC) curves were constructed to determine the area under the curve (AUC) of SUA and urinary uric acid excretion indicators for detecting the risk of EIKF. Confidence intervals (CIs) of 95% were used. A *P*-value < 0.05 was considered statistically significant. All statistical analyses were performed using IBM SPSS statistical software version 22 for Windows (IBM Corp, Armonk, New York, USA).

## Results

### Clinical characteristics of the study subjects

General clinical data are shown in Table 1. A total of 628 patients were included, with median age as 55 years old, ranging from 18 to 90 years, and 62.7% of the patients were males. The median BMI of the patients was 23.9 kg/m^2^ (range 14.2–38.4 kg/m^2^). The proportion of patients with a history of hypertension and alcohol consumption was 31.4% and 12.9%, respectively. The number of patients with diabetic ketoacidosis was 127, accounting for 20%. The median HbA1c and HOMA-IR was 11.8% (range 5%–19.9%) and 1.507 (range 1.500−1.550), respectively. The median SUA and Uur was 296 µmol/L and 3,587 µmol/24 h, respectively. The comparisons of clinical and laboratory indicators between EIKF group and non-EIKF group are also presented in Table 1. EIKF group and non-EIKF group significantly differed in age, systolic blood pressure (SBP), history of hypertension, SUA, UACR, FEur, Cur, FLur, EurGF, Uur, HOMA-β, and HOMA- IR (*P* < 0.05).

**Table 1:** Clinical characteristics of subjects.

Characteristics	All (*n* = 628)	Non-EIKF (*n* = 369)	EIKF (*n* = 259)	*P* value
Demographics				
Age, years	55 (47, 64)	52 (44, 59)	60 (51, 68)	0.000^#^
Male, n (%)	394 (62.7)	239 (64.8)	155 (59.8)	0.209
BMI, kg/m^2^	23.9 (21.6, 26.2)	23.7 (21.4, 26.4)	23.9 (21.7, 26.2)	0.494
Smoking, n (%)	175 (27.9)	114 (30.9)	61 (23.6)	0.043^*^
Drinking, n (%)	81 (12.9)	52 (14.1)	29 (11.2)	0.287
Hypertension, n (%)	197 (31.4)	83 (22.5)	114 (44.0)	0.000
SBP, mmHg	133 (121, 145)	131 (120, 143)	135 (124, 151)	0.001^#^
DBP, mmHg	85 ± 12	85 ± 11	86 ± 12	0.276
DKA, n (%)	127 (20.2)	81 (22.0)	46 (17.8)	0.198
Laboratory measurements				
HbA1c, %	11.8 (10.2, 13.4)	12.0 (10.4, 13.8)	11.6 (10.0, 13.2)	0.039^#^
FBG, mmol/L	10.98 (8.02, 13.97)	11.29 (8.00, 13.92)	10.78 (8.07, 14.07)	0.636
Fasting C-Peptide, μg/L	1.80 (1.25, 2.33)	1.62 (1.12, 2.23)	1.92 (1.38, 2.39)	0.001^#^
HOMA-β	0.063 (0.039, 0.113)	0.058 (0.037, 0.105)	0.071 (0.045, 0.119)	0.010^*^
HOMA-IR	1.507 (1.504, 1.510)	1.506 (1.504, 1.510)	1.508 (1.505, 1.511)	0.009^*^
TG, mmol/L	1.41 (1.04, 2.01)	1.39 (1.03, 1.98)	1.45 (1.06, 2.10)	0.439
TC, mmol/L	4.87 (4.18, 5.71)	4.92 (4.24, 5.80)	4.77 (4.07, 5.57)	0.119
LDL-C, mmol/L	3.20 (2.59, 3.96)	3.25 (2.62, 4.05)	3.15 (2.51, 3.87)	0.111
HDL-C, mmol/L	1.02 (0.86, 1.88)	0.86 (0.73, 1.02)	0.89 (0.76, 1.02)	0.963
ApoA1, g/L	1.15 (1.01, 1.31)	1.15 (1.00, 1.30)	1.14 (1.02, 1.31)	0.835
ApoB, g/L	1.05 (0.87, 1.24)	1.07 (0.88, 1.29)	1.03 (0.85, 1.19)	0.006^*^
SUA, μmol/L	296 (240, 340)	286 (228, 334)	309 (261, 348)	0.001^#^
SCr, μmol/L	70 (58, 82)	63 (55, 74)	83 (69, 92)	0.000^#^
eGFR, ml/min/1.73 m^2^	98.75 (84.55, 115.54)	110.46 (99.02, 125.89)	81.56 (71.34, 88.43)	0.000^#^
Ccr, ml/min	95.44 (71.84, 123.08)	105.89 (83.82, 134.39)	80.45 (60.90, 104.32)	0.000^#^
UACR, mg/gCr	9 (5, 21)	8 (5, 12)	25 (6, 61)	0.000^#^
24 h urine biochemistry				
UV, L/24 h	2.1 (1.6, 2.8)	2.2 (1.6, 2.9)	2.1 (1.5, 2.8)	0.156
Ucr, μmol/24 h	9,484.0 (7,179.5, 12,072.9)	9,525.1 (7,293.5, 12,522.2)	9,369.8 (6,838.0, 11,432.0)	0.145
Urinary total protein, mg/24 h	139.5 (94.6, 222.9)	133.0 (92.4, 215.0)	149.4 (98.0, 245.0)	0.081
Urinary uric acid excretion indicators				
Uur, μmol/24 h	3,587 (2,811, 4,450)	3,707 (2,833, 4,676)	3,413 (2,766, 4,230)	0.011^*^
FEur, %	8.99 (7.16, 11.89)	8.45 (6.92, 11.05)	9.85 (7.53, 13.15)	0.000^#^
Cur, ml/min	8.79 (6.54, 11.43)	9.56 (7.02, 12.04)	8.16 (6.22, 10.27)	0.000^#^
FLur, μmol/min	26.50 (19.24, 36.50)	28.46 (20.54, 40.46)	23.81 (16.42, 33.01)	0.000^#^
EurGF, μmol/L	25.75 (21.38, 30.86)	24.30 (20.12, 27.91)	28.85 (23.86, 36.40)	0.000^#^
Uur/Ucr, μmol/µmol	0.38 (0.31, 0.46)	0.38 (0.32, 0.45)	0.37 (0.30, 0.47)	0.406

**Notes.**

Continuous data were expressed as mean (standard deviation) or median (interquartile range), categorical data as n (%).

* *P*-value < 0.05.

# *P*-value < 0.0 0 5.

Abbreviations EIKF early impaired kidney function BMI body mass index BP blood pressure DKA diabetic ketoacidosis HbA1c glycated hemoglobin FPG fasting plasma glucose TG triglyceride TC total cholesterol LDL-C low-density lipoprotein cholesterol HDL-C high-density lipoprotein-cholesterol Apo apolipoprotein SUA serum uric acid e GFR estimated glomerular filtration rate SCr serum creatinine UACR urinary albumin creatinine ratio Ccr creatinine clearance UV urine volume Ucr urine creatinine Uur 24 h urinary excretion of uric acid Cur uric acid clearance rate FEur fractional excretion of uric acid FLur glomerular filtration load of uric acid EurGF excretion of uric acid per volume of glomerular filtration Ucr urine creatinine

### Correlation analysis of SUA, uric acid excretion indactors and EIKF

**Figure 1:**
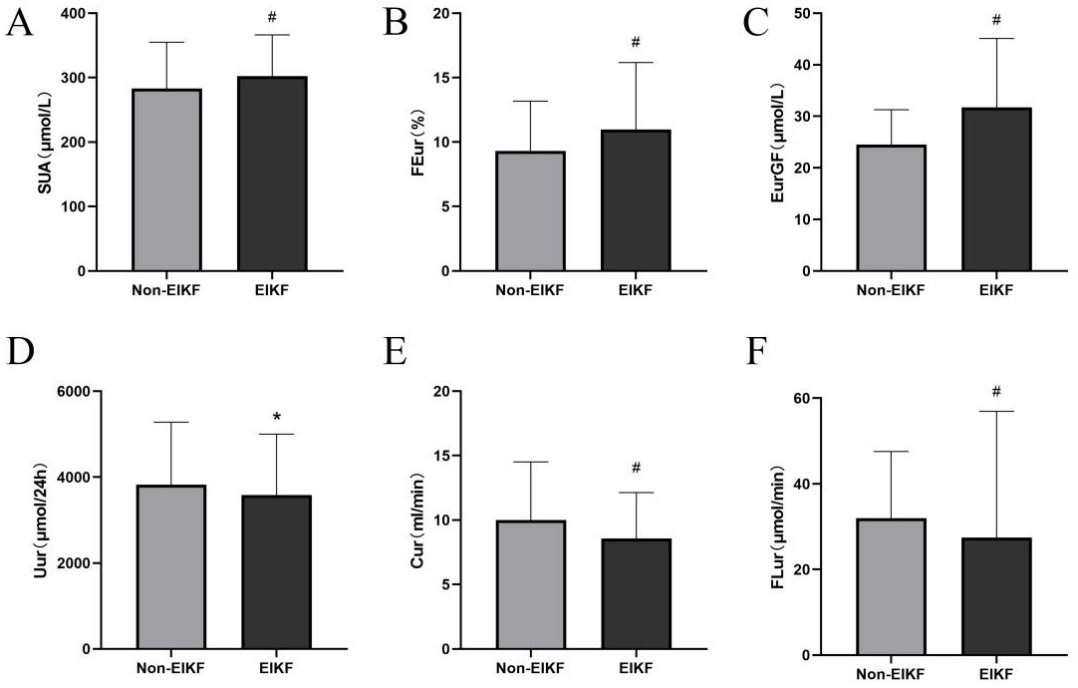
Comparison of SUA (A), FEur (B), EurGF (C), Uur (D), Cur (E) and FLur (F) between EIKF group and non-EIKF group in patients with newly diagnosed T2DM. *P-value < 0.05. # *P*-value < 0.0 05. Abbreviations: EIKF, early impaired kidney function; SUA, serum uric acid; FEur, fractional excretion of uric acid; EurGF, excretion of uric acid per volume of glomerular filtration; Uur, 24 h urinary excretion of uric acid; Cur, uric acid clearance rate; FLur, glomerular filtration load of uric acid.

SUA, FEur and EurGF increased (all *P* < 0.005, Figs. 1A–1C), while Uur, Cur and FLur decreased in EIKF group (all *P* < 0.05, Figs. 1D–1F), compared with non-EIKF group. Spearman correlation analysis showed that age, SBP, HOMA-β, HOMA-IR, SUA, FEur and EurGF negatively correlate with eGFR level (*P* < 0.05), while SBP, Uur, Cur and FLur positively correlate with eGFR level (*P* < 0.05). Uur, Cur and FLur positively correlate with 24-hour urinary protein levels (*P* < 0.05, Table 2).

**Table 2:** Factors correlated with eGFR and urinary total protein.

Variable	eGFR		Urinary total protein	
	Correlation coefficient	*P* value	Correlation coefficient	*P* value
Age	−0.424	0.000^#^	−0.015	0.711
SBP	−0.134	0.001^#^	0.101	0.012^*^
HbA1c	0.062	0.120	0.072	0.070
Fasting C-Peptide	−0.136	0.001^#^	−0.051	0.201
HOMA-β	−0.089	0.026^*^	0.005	0.907
HOMA-IR	−0.114	0.004^#^	−0.069	0.085
ApoB	0.074	0.064	−0.046	0.250
SUA	−0.143	0.000^#^	−0.026	0.255
Ccr	0.452	0.000^#^	0.225	0.000^#^
Uur	0.101	0.011^*^	0.333	0.000^#^
FEur	−0.248	0.000^#^	0.075	0.059
Cur	0.186	0.000^#^	0.306	0.000^#^
FLur	0.274	0.000^#^	0.177	0.000^#^
EurGF	−0.462	0.000^#^	0.055	0.171

**Notes.**

* *P*-value < 0.05.

# *P*-value < 0.0 0 5.

Abbreviations SBP systolic blood pressure HbA1c glycated hemoglobin Apo apolipoprotein SUA serum uric acid e GFR estimated glomerular filtration rate Uur 24 h urinary excretion of uric acid Cur uric acid clearance rate FEur fractional excretion of uric acid FLur glomerular filtration load of uric acid EurGF excretion of uric acid per volume of glomerular filtration

**Figure 2:**
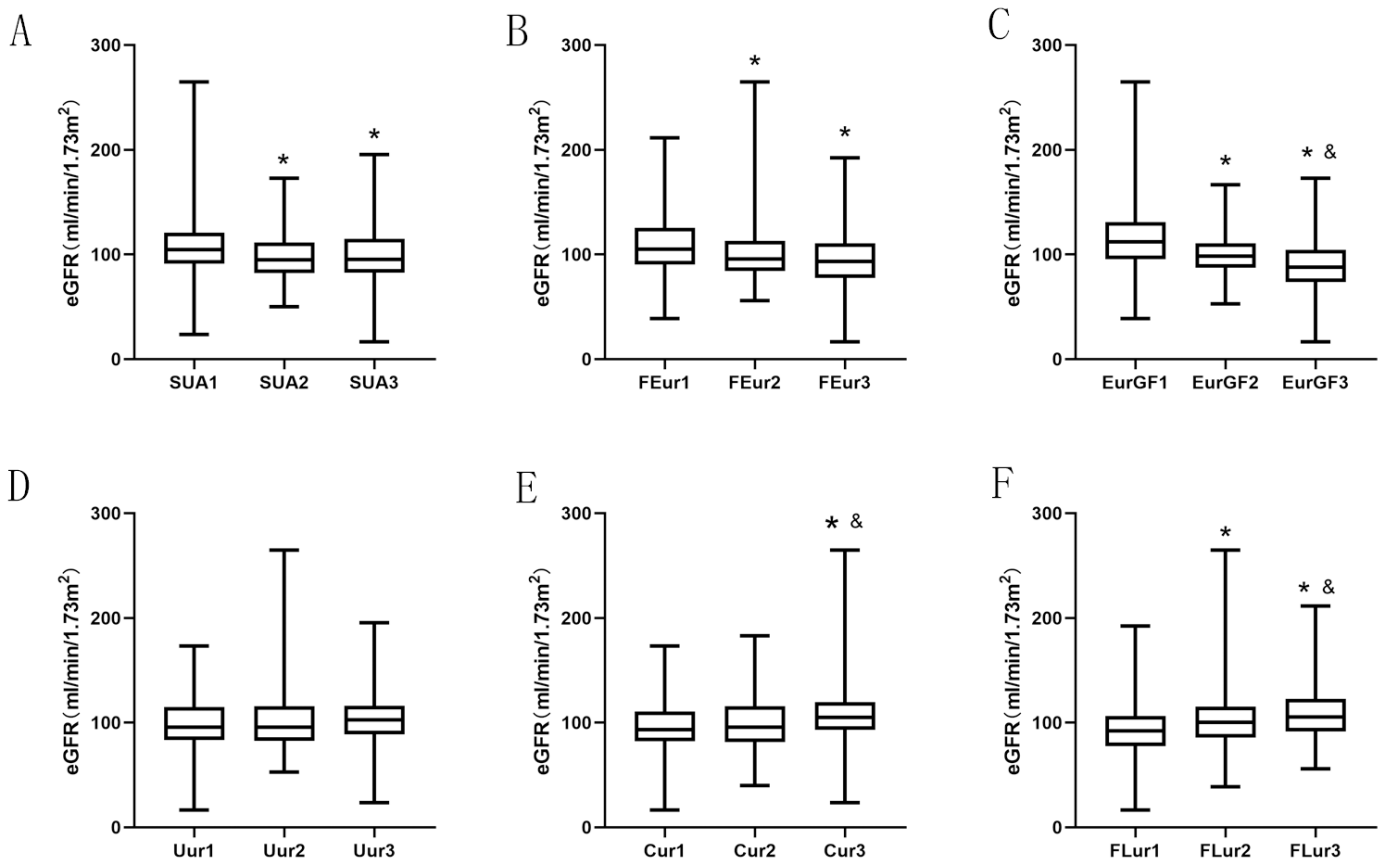
Levels of eGFR in different SUA (A), FEur (B), EurGF (C), Uur (D), Cur (E) and FLur (F) tertiles. *P-value < 0.05 tertiles 1 *vs.* tertiles 2 and tertiles 3; & *P*-value < 0.0 5. tertiles 2 *vs* tertiles 3. Abbreviations: e GFR, estimated glomerular filtration rate; SUA, serum uric acid; FEur, fractional excretion of uric acid; EurGF, excretion of uric acid per volume of glomerular filtration; Uur, 24 h urinary excretion of uric acid; Cur, uric acid clearance rate; FLur, glomerular filtration load of uric acid.

SUA and uric acid excretion indactors including FEur, EurGF, Uur, Cur, and FLur, were stratified by tertiles (Fig. 2). The eGFR levels in the higher and medium SUA ,FEur and EurGF groups were significantly decreased compared with low SUA group (*P* < 0.05, Figs. 2A–2C). The eGFR in Cur 3 group was significantly increased compared with Cur 1 group and Cur 2 group (*P* < 0.05, Fig. 2E), and the eGFR in FLur 3 group and FLur 2 group was significantly increased compared with FLur 1 group (*P* < 0.05, Fig. 2F). No significant difference was observed in eGFR levels among Uur groups (*P* > 0.05, Fig. 2D).

**Figure 3:**
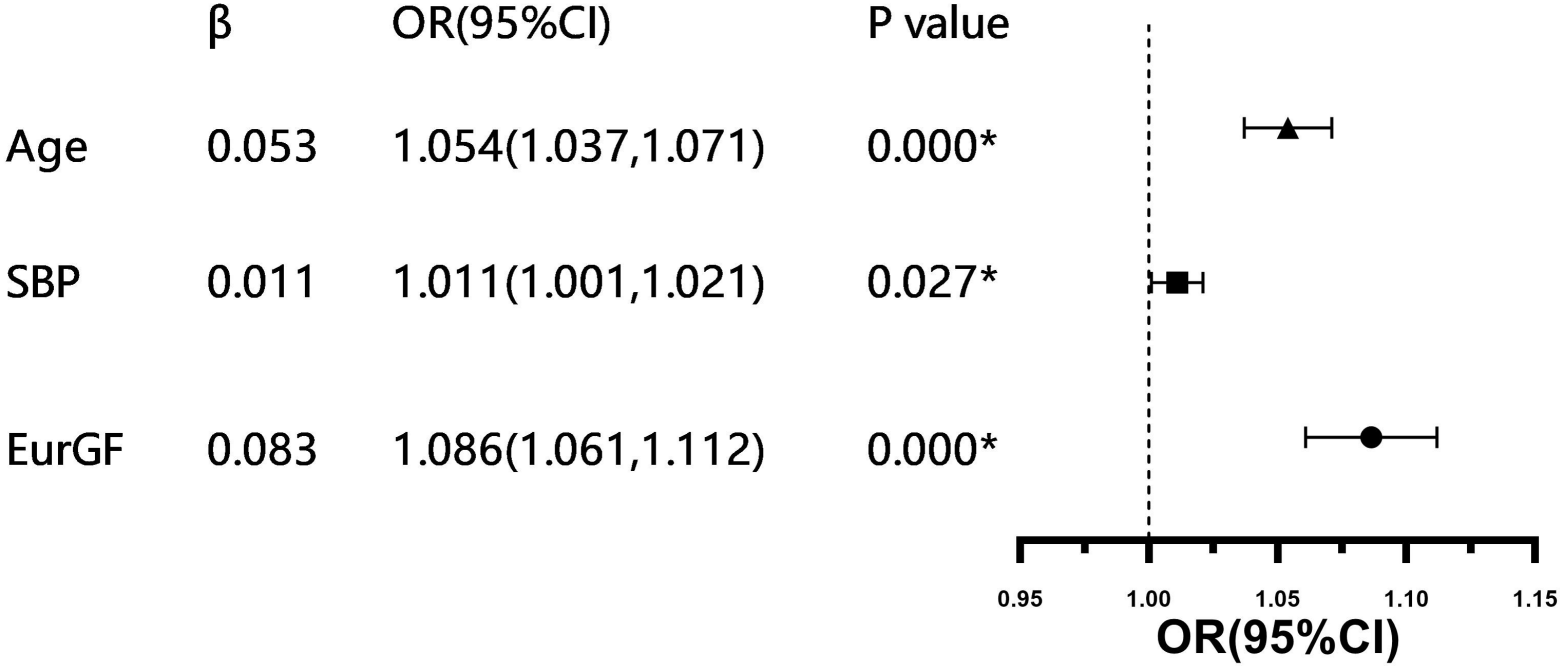
Forest map of multivariate logistic regression analysis of associated factors of EIKF in patients with newly diagnosed T2DM. *P-value < 0.05. Abbreviations: SBP, systolic blood pressure; EurGF, excretion of uric acid per volume of glomerular filtration.

**Figure 4:**
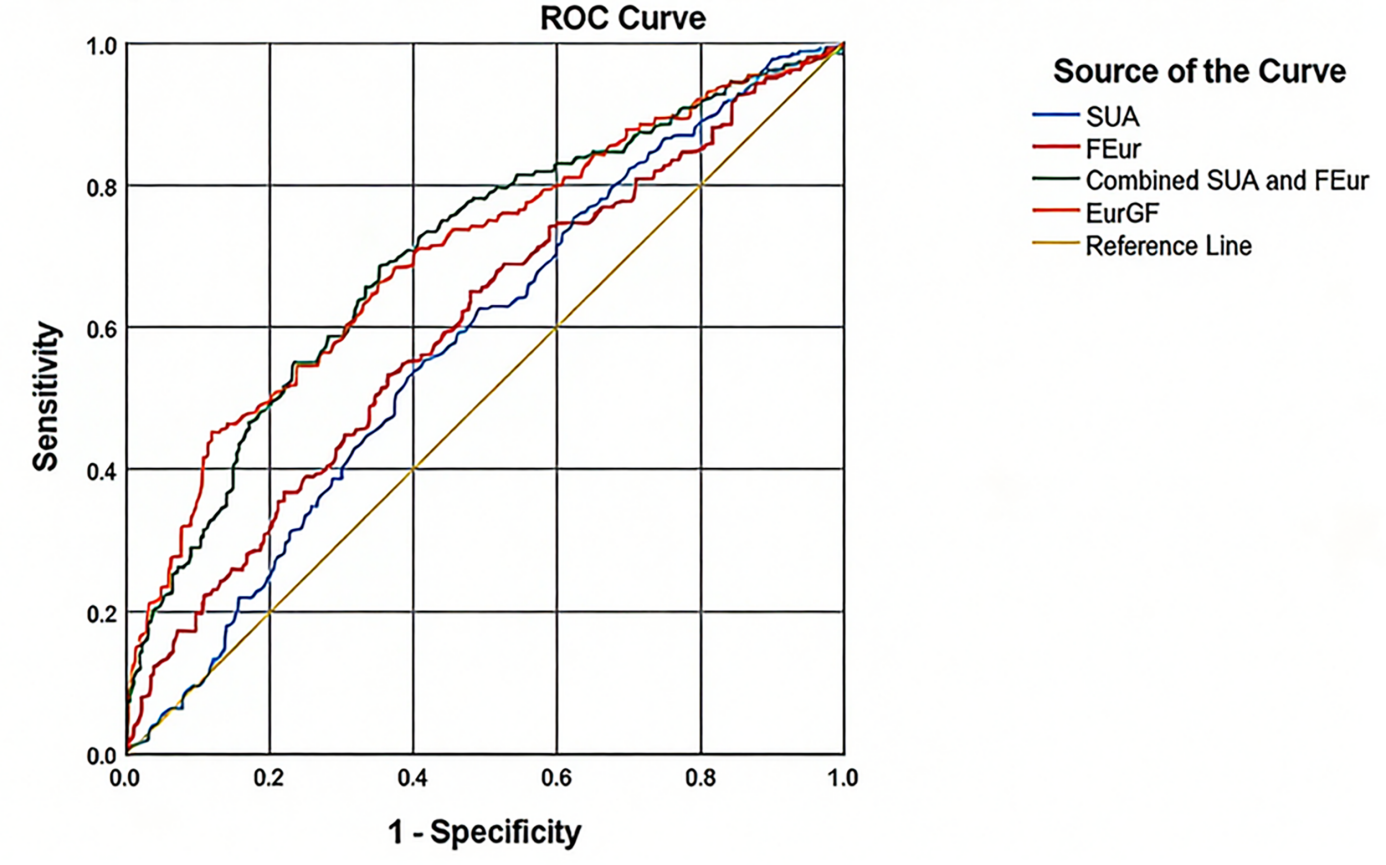
Receiver operator characteristic (ROC) curve of SUA, FEur and EurGF for detecting the potential risk of EIKF in patients with newly diagnosed T2DM. Abbreviations: SUA, serum uric acid; FEur, fractional excretion of uric acid; EurGF, excretion of uric acid per volume of glomerular filtration.

Multivariate logistic regression analysis revealed that age (OR (95% CI) 1.054 (1.037,1.071), *P* < 0.001), SBP (OR (95% CI), 1.011 (1.001,1.021), *P* < 0.05) and EurGF (OR (95% CI), 1.086 (1.061,1.112), *P* < 0.001) significantly correlate with the presence of EIKF (Table 3, Fig. 3). The results of the ROC curve analysis are presented in Fig. 4. The cut-off value for detecting the presence of EIKF is 300.5 µmol/l for SUA (55.6% sensitivity, 58.3% specificity, and AUC = 0.579 ± 0.048), 8.6% for FEur (64.9% sensitivity, 52% specificity, and AUC = 0.601 ± 0.045), and 30.73 µmol/L for EurGF (45.2% sensitivity, 88.1% specificity, and AUC = 0.701 ± 0.042). The combination of SUA and FEur has a high detective efficiency, with an AUC of 0.701 (95% CI [0.659–0.743]) (*P* = 0.000), a sensitivity of 68.7%, and a specificity of 64.5%.

**Table 3:** Multivariate logistic regression analysis of associated factors of EIKF in patients with newly diagnosed T2DM.

	β	OR (95% CI)	*P* value
Age	0.053	1.054 (1.037, 1.071)	0.000^*^
SBP	0.011	1.011 (1.001, 1.021)	0.027^*^
EurGF	0.083	1.086 (1.061, 1.112)	0.000^*^
HOMA-IR	29.411	5.93E+12 (0.001, 5.77E+28)	0.117

**Notes.**

* *P*-value < 0.05.

Abbreviations SBP systolic blood pressure EurGF excretion of uric acid per volume of glomerular filtration

## Discussion

Studies have demonstrated that SUA is associated with the development and progression of chronic renal failure. However, further research is required to determine whether a causal relationship exists between SUA and the onset of chronic renal impairment (Johnson et al., 2013). Recently, an increasing number of studies have focused on the relationship between uric acid excretion and kidney disease. Evidence indicates that in patients with chronic kidney disease (CKD), uric acid excretion indices such as FEur and uric acid clearance (Cur) are positively correlated with diastolic and mean blood pressure (Xu et al., 2020). In non-diabetic patients with stage CKD1-2, estimated uric acid glomerular filtration (EurGF) is closely associated with urine glucose (Feng et al., 2021). In diabetic patients, urinary uric acid (Uur) is positively correlated with estimated glomerular filtration rate (eGFR) (Li et al., 2015). However, the association between uric acid excretion indices and EIKF in newly diagnosed T2DM patients with SUA within the normal range has not been previously investigated. In this study, we found that SUA and uric acid excretion indices (fractional excretion of uric acid (FEur) and EurGF) have certain diagnostic value for detecting EIKF in newly diagnosed T2DM patients with normal SUA levels. The combination of SUA and urinary uric acid excretion indices demonstrated greater diagnostic efficacy.

Hyperuricemia is recognized as one of the risk factors for the development of metabolic syndrome and diabetes (Paul, Anoopkumar & Krishnan, 2017). Among newly diagnosed T2DM patients, elevated uric acid levels are also associated with hypertension and obesity (Singh et al., 2023c; Singh et al., 2023a). Furthermore, hyperuricemia is a risk factor for the development of microalbuminuria and macroalbuminuria (Singh et al., 2023b). Numerous studies have indicated that elevated SUA may predict the onset of CKD in T2DM patients, although conflicting results exist (Ceriello et al., 2017). Additionally, a prospective cohort study involving 2,042 CKD patients showed that the use of allopurinol and febuxostat did not confer renal protective effects in hyperuricemic individuals (Oh et al., 2019). Although elevated SUA levels are linked to the progression of chronic kidney disease, uric acid-lowering therapy has not demonstrated meaningful protective effects on kidney function in patients with stage 3 or 4 CKD (Badve, 2020) or in those with type 1 diabetes mellitus (T1DM) and early-to-moderate diabetic kidney disease (Doria, 2020). These findings align with previous reports of a U-shaped association between SUA levels and the decline in kidney function (Seguro et al., 2015). A study in rural Korean men revealed that both higher and lower serum uric acid levels are associated with the incidence of CKD (Mun et al., 2018). Another large-scale study involving 138,511 participants indicated that both elevated and reduced SUA levels are risk factors for CKD development (Nakayama et al., 2021). Our study demonstrated that higher SUA is associated with EIKF in newly diagnosed T2DM patients, with SUA > 300.5 µmol/L identified as the optimal cutoff value for detecting EIKF. This suggests that high-normal SUA is a correlating factor for EIKF in this population. Notably, according to guidelines for gout management, maintaining SUA below 300.5 µmol/L is also a therapeutic target for patients with tophus (Dalbeth, Gaffo & Abhishek, 2021; Keller & Mandell, 2022).

Several basic science studies have reported that uric acid may contribute to CKD through multiple mechanisms, including genetic predisposition (Xie et al., 2013), oxidative stress (Sánchez-Lozada et al., 2012), endothelial dysfunction (Rabadi et al., 2012), renin-angiotensin system (RAS) activation (Corry et al., 2008; Kang et al., 2005), and inflammation (Ryu et al., 2013). Under physiological conditions, approximately two-thirds of SUA is excreted by the kidneys, while the remaining one-third is eliminated *via* the gastrointestinal tract. Although 99% of uric acid is filtered through the glomerulus, about 90% is reabsorbed by the proximal renal tubules. Simultaneously, uric acid is secreted in the proximal tubules and the intestine *via* specific transporters, primarily members of the organic anion transporter (OAT) family (OAT1–4), ABCG2, and GLUT9 (Lipkowitz, 2012). However, the precise molecular mechanisms require further investigation. Cross-sectional analyses of data from the National Health and Nutrition Examination Survey (NHANES) suggest that renal glomerular function plays a more critical role in regulating uric acid homeostasis than previously recognized (Steinman & Krishnan, 2012). Theoretically, diabetic patients with poor glycemic control exhibit worse kidney function (Bo et al., 2001). Recent studies have also shown that sodium-glucose cotransporter-2 (SGLT2) inhibitors increase urinary glucose excretion by modulating SGLT2 activity in diabetic patients, thereby lowering blood glucose and simultaneously altering uric acid transport activity in renal tubules (Chino et al., 2014). Thus, uric acid excretion participates in blood glucose regulation through renal tubular uric acid transporters. In the present study, no significant negative correlation was observed between blood glucose, islet function, and kidney function, which may be attributed to the relatively short duration of diabetes in these patients. It is well established that increasing age is an important factor in the development of hyperuricemia and chronic kidney disease. Li et al. (2022) demonstrated that when advanced age and hyperuricemia coexist, their combined effect on CKD progression exceeds the sum of their individual effects. A recent nationwide study across 31 provinces in China showed that the risk of gout in men decreases with age, whereas gout incidence in women over 50 years increases significantly compared to those under 50, possibly due to the uricosuric effects of estrogen (Song et al., 2022). In our study, the EIKF group was 8 years older on average than the non-EIKF group, and the proportion of females was numerically higher.

Increasing evidence confirms that SUA is associated with renin levels and hypertension (Paul, Anoopkumar & Krishnan, 2017). An Indian study showed that in patients with newly diagnosed diabetes, high blood pressure and elevated uric acid levels are positively correlated (Singh et al., 2023c). Our study revealed that SBP was negatively correlated with eGFR and positively correlated with 24-hour urinary total protein. A recent study by Li et al. (2018) demonstrated that uric acid excretion is associated with urinary sodium and potassium levels in hypertensive patients with impaired renal function, further supporting the complex interplay between hypertension, hyperuricemia, and renal function (Guan et al., 2020; Mallat et al., 2016). In this study, we excluded patients receiving long-term angiotensin II receptor blocker (ARB) therapy to minimize potential confounding effects on uric acid excretion and kidney function.

Uur, Cur, FEur, FLur, and EurGF are the primary indicators of renal uric acid excretion, aiding in the differentiation between reduced excretion and overproduction (Indraratna et al., 2010). Decreased uric acid excretion is detrimental to the cardiovascular, cerebrovascular, renal, and other systems. Impaired renal function reduces urinary uric acid excretion and increases gout risk (Bardin & Richette, 2017). A recent cross-sectional study showed that reduced Uur is one of the factors contributing to elevated SUA in patients with proteinuria (Zou et al., 2015). Traditionally, albuminuria has been considered a consequence of diabetes-induced glomerular injury. However, it is now increasingly recognized that the renal tubulointerstitium also contributes to the pathogenesis of diabetic nephropathy. Although diabetic nephropathy is classically described as a glomerulopathy, tubular dysfunction is believed to occur earlier in the disease process. Recent studies have consistently shown that markers of proximal tubular damage are closely associated with diabetic kidney injury (Agarwal, Sarma & Devi, 2018; Takagi et al., 2024). Our study demonstrated that Uur was reduced in patients with early renal impairment and decreased progressively with declining eGFR. Cur and FLur exhibited similar trends, suggesting that uric acid excretion indices (Uur, Cur, FLur) are diminished in T2DM patients with early renal dysfunction. Studies indicate that Cur and FLur are valuable for monitoring uric acid excretion in patients with renal insufficiency (Kannangara et al., 2012; Guan et al., 2020), with FLur being more sensitive to variations in creatinine and SUA. Compared to random urine samples, Uur is more accurate but is influenced by diet, urine volume, kidney function, and circadian rhythm (Vgontzas et al., 2000). Therefore, Cur and FLur may be more suitable than Uur for evaluating uric acid excretion patterns in newly diagnosed T2DM patients. Regarding proteinuria, three uric acid excretion indices (Uur, FLur, and Cur) were positively correlated with 24-hour urinary total protein, whereas SUA, FEur, and EurGF did not show significant associations in this study. However, in another study involving T2DM patients, SUA was positively correlated with proteinuria (Liang et al., 2016). A clinical trial on URAT1-targeted uric acid (UA)-lowering drugs showed that CKD patients treated with UA-lowering agents exhibited increased urinary uric acid excretion, reduced SUA, and significantly improved proteinuria and renal function (Yanai et al., 2023). This discrepancy may be explained by the fact that our study population consisted of newly diagnosed T2DM patients, in whom the mechanism of renal uric acid excretion may not yet be linked to proteinuria in early renal damage. It may also reflect the use of random 24-hour urine samples without dietary restriction.

A previous study in Chinese CKD subjects revealed that patients in CKD stage 5b exhibited higher levels of FEur and EurGF, and lower levels of Uur, Cur, and FLur (Jing et al., 2018). There may be four explanations for the changes of these parameters. The first one is the compensation of residual nephrons to delay kidney damage. The second one is the abnormal uric acid metabolism of uremic patients themselves. The third one is the abnormal secretion of renal tubules. And the fourth is the compensatory expression of ABCG2 or SLC2A9 genes in the gut in patients with CKD. Population-based genetic studies have shown that ABCG2 or SLC2A9 genes are associated with uric acid excretion in CKD (Guan et al., 2020). Also, population-based genetic studies have indicated that ABCG2 or SLC2A9 genes are associated with uric acid excretion in CKD (Jing et al., 2018). This is consistent with the results of this study. The higher FEur and EurGF, the higher incidence of early kidney damage in newly diagnosed T2DM patients. The results indicated that EIKF in newly diagnosed T2DM patients may have some compensation, since FEur is the ratio between renal uric acid clearance and creatinine clearance. Multiple studies have shown that EurGF can better reflect renal tubule excretion of uric acid since it eliminates the impact of urine volume and creatinine clearance (Kannangara et al., 2012; Ma et al., 2016).

In this study, SUA, FEur, and EurGF demonstrated significant value in identifying early renal damage in newly diagnosed T2DM patients, with EurGF emerging as a relatively robust indicator. Therefore, early renal damage may not solely result from abnormal renal tubular secretion but may also involve altered expression of intestinal uric acid transporters such as ABCG2. However, further experimental research is needed. In terms of calculation, EurGF equals SUA × FEur, and our study confirmed that the combined use of SUA and FEur provides greater diagnostic efficacy for early kidney damage in newly diagnosed T2DM patients, comparable to EurGF.

The present study has several potential limitations. First, as noted, the study involved a limited number of participants from a single center, and as it was conducted among hospitalized T2DM patients, it remains uncertain whether similar conclusions apply to other populations. Second, all outcomes were derived from retrospective analysis. Changes in SUA parameters may represent early correlates of mild or established kidney injury, but a cause-and-effect relationship between EIKF and SUA parameters cannot be established. Third, since EIKF is defined as eGFR < 90 mL/min or urine albumin-to-creatinine ratio (UACR) ≥ 30 mg/g, it may not reflect definitive structural renal damage. Therefore, results should be interpreted with caution. Nevertheless, to the best of our knowledge, this is the first study to investigate the association between SUA, urinary uric acid excretion indices, and EIKF in patients with newly diagnosed T2DM.

## Conclusion

In conclusion, this study indicates that among newly diagnosed T2DM patients with normal SUA levels, elevated SUA, FEur, and EurGF have clinical significance in detecting early renal damage. Notably, EurGF emerges as a relatively robust indicator for identifying individuals at increased risk of EIKF.

##  Supplemental Information

10.7717/peerj.20881/supp-1 Supplemental Information 1 Raw data

10.7717/peerj.20881/supp-2 Supplemental Information 2 Categorical data codebook

## Additional Information and Declarations

### Data Availability

The following information was supplied regarding data availability: The raw measurements are available in the Supplemental File.

### Clinical Trial Registration

The following information was supplied regarding Clinical Trial registration: 2023-hg-ks-49.
